# A New System of Cure

**Published:** 1891-02

**Authors:** 


### A NEW SYSTEM OF CURE.

Half a century ago Sebastian Kneipp, the village priest of Voerishofen, Bavaria, was very ill. The doctors said he must surely die. Then it was that the priest invented a system of self-cure, which speedily restored him to entire health. He has since devoted his life to developing and perfecting his system. He began by curing himself. Now he cures others. The little village is crowded with people, who come from near and far to take his advice, which is given gratis.

Father Kneipp does not believe in wearing wool or flannel next to the skin; he declares that it renders the skin delicate, and his great aim is to harden and invigorate—not, be it observed, by violent means, which he strongly deprecates, but by natural and gradual ones. He recommends that all underclothing be made of very coarse linen, the roughness of which stimulates the skin without enervating it, as wool does, and, moreover, possesses the advantage of allowing the perspiration to pass through it quickly. Wool, he says, often induces rheumatism, and is only advisable for outer clothes. Water plays an important part in Father Kneipp's system; but his mode of water-cure differs greatly from that usually known under the name of hydropathy. He prefers cold to warm water; but employs it cautiously, and allows old or nervous persons to use tepid water. Before every thing he enjoins rapidity in bathing. According to him, a cold bath, including undressing and dressing, should only last five minutes. This seems an impossible period in which to take a bath. It is, however, explained in the next and one of the most startling rules in the Kneipp method: the patient is forbidden to attempt to dry himself after the bath, but is told to put his coarse linen underclothes straight on to his wet body, then his outer clothes, and then to take at least a quarter of an hour's exercise. Father Kneipp declares that the drops of water left on the skin serve as fuel for the inner warmth, which uses them as material to form a rapid and intense glow of heat all over the body, assisted by the activity of the skin induced by the coarseness of the linen in contact with it.

Another means of hardening and invigorating the body and promoting circulation adopted by Father Kneipp is the practice of walking or running barefooted in wet grass, in cold weather, or in freshly fallen snow. Voerishofen lies in a valley, in the midst of green meadows, which seem to have been made especially for this form of exercise, and are constantly occupied by the patients taking their daily runs with naked feet. The exercise at first lasts only five minutes, but the period is gradually increased to half an hour. At the end of the prescribed time, the patient is ordered to put on dry socks (made of coarse yarn, precisely similar to that of which the linen for the underclothing is manufactured) without drying his feet, then his boots, and then take a smart walk.

Father Kneipp fulminates furiously against the amount of tea and coffee drunk by the present generation, to which indulgence he attributes the enormous prevalence of nervousness and nervous diseases. He also objects to the great quantity of meat usually consumed, the proportion of which, in relation to other foods, he considers far too large. The nourishment he recommends consists chiefly of bread, fruit, vegetables and milk. He particularly praises the many farinaceous dishes, and dishes composed wholly of vegetables peculiar to Viennese cookery, and little known elsewhere. He strongly recommends brown bread, for which he gives a receipt specially adapted for dyspeptic patients. His two particular "fancies" in the way of food, those which he considers the healthiest and most nourishing, are peas and sauerkraut! There are few better meals, he says, than plenty of fresh fruit and a piece of bread. Three meals a day, he maintains, are sufficient. If more are taken the stomach has not time to recover from one process of digestion before it is called upon to begin another. The more moderately a man eats the more chance he has of keeping his digestive organs in good order and retaining them so to old age. He advises his patients to drink before eating, never while eating, and after eating only if very decided thirst be felt; and then but moderately. He advocates hard beds, and cool, well-ventilated bedrooms. He approves of the use, but not the abuse, of all good things except tea and coffee, which he does not consider good at all. He is much looked up to by the medical profession, and many doctors go to Voerishofen to study his method.

